# Open Water Swimming: Swimmers’ Kinematical and Neuromuscular Characterisation in 5 km Swim

**DOI:** 10.3390/sports13100335

**Published:** 2025-10-01

**Authors:** Ana Conceição, Daniel Marinho, Jan Stastny, Carlos Gonçalves, João Freitas, Renato da Costa-Machado, Hugo Louro

**Affiliations:** 1 Department of Sport Sciences, Sport Sciences School of Rio Maior, Polytechnic Institute of Santarém, 2040-413 Santarém, Portugal; joaofreitas@esdrm.ipsantarem.pt (J.F.); renato.machado@esdrm.ipsantarem.pt (R.d.C.-M.); hlouro@esdrm.ipsantarem.pt (H.L.); 2 Research Center in Sports Sciences, Health Sciences and Human Development (CIDESD), 6201-001 Covilhã, Portugal; marinho.d@gmail.com; 3 Department of Sport Sciences, University of Beira Interior, 6201-001 Covilhã, Portugal; 4 Centre of Sports Activities, Brno University of Technology, 616 69 Brno, Czech Republic; jan.stastny@vut.cz; 5 Department of Sport Sciences, Faculty of Sports Sciences of Cáceres, University of Extremadura, 1003 Cáceres, Spain; 210500001@esdrm.ipsantarem.pt

**Keywords:** biomechanics, open water swimming, surface electromyography, freestyle

## Abstract

This study aimed to characterize and analyse the kinematic parameters and muscle activity of swimmers in open water swimming (OWS). Nine male swimmers (age: 25.4 ± 11.9 years; body mass: 75.9 ± 9.0 kg; height: 180.7 ± 6.7 cm; and arm span: 185.6 ± 10.3 cm) were evaluated in an open environment (lake), performing 5 m × 1000 m at maximum intensity, with a rest of 30 s every 1000 m. For kinematical analyses, the stroke rate (SR), swimming velocity (v), stroke length (SL), and stroke index (SI) were calculated. Surface EMG data were recorded in seven muscles—upper trapezius (UP); latissimus dorsi (LD); pectoralis major (PM); posterior deltoid (PD); anterior deltoid (AD); triceps brachii (TB); and biceps brachii (BB)—for the underwater and recovery phases of the stroke. SL (F = 3.41, *p* = 0.06, η^2^ = 0.30) and SI (F = 3.29, *p* = 0.08, η^2^ = 0.29) changed along the covered distances, and SR (F = 1.54, *p* = 0.24, η^2^ = 0.16) increased, especially in the last 1000 m (32.5 ± 3.0 cycles-min^−1^). AD was highly activated in recovery, showing statistical differences from the beginning (*p* ≤ 0.01) to the end of the race (*p* = 0.03). The TB muscle was mostly recruited in the underwater phase, from the start (*p* ≤ 0.01) to the finish (*p* = 0.03), showing a significant difference in each lap, with a large effect. LD showed significant differences in muscle activation, from 1000 m (*p* ≤ 0.01) with a huge effect, to 5000 m (*p* ≤ 0.01), with a large effect. These results suggested that the UT and AD muscles had higher activity in recovery than the underwater phase, and TB and LD were higher in the underwater phase.

## 1. Introduction

The World Aquatics defines open water swimming (OWS) as any competition that takes place in rivers, lakes, oceans, or water channels, as the dynamics of the environment that swimmers may encounter are very varied [1,2]. The resurgence of OWS as one of the most recent disciplines of swimming has aroused the curiosity of coaches, swimmers, and all of those involved in the process. The ecological differences between swimming in a pool and swimming in the sea lead to constraints that impose difficulties (weather conditions, water typology, currents, temperature, and navigation in the courses) for some swimmers and ease for others [1,2,3].

The higher challenge in OWS research is creating standardised study conditions: it is almost impossible to reproduce the situational challenges of an open water event in controlled laboratory conditions, because open water events may be characterised by extreme environmental conditions that impact the overall performance [1]. OW swimmers can adapt to different environmental conditions and their opponents’ race strategies, so this event can be considered an open-skill sport compared to pool swimming, where the swimmers would make adjustments in their swimming technique, namely, in biomechanical and physiological factors [3].

However, the research on OWS is increasing, with some studies focused on the physiological [2,4,5,6,7] and biomechanical factors, to better understand the muscular and kinematic parameters [8,9,10]; however, knowledge on neuromuscular analysis in open water swimmers in a natural context remains non-existent.

The front crawl technique, where breathing is unilateral, causes measurable differences in factors that are known to significantly impact performance, namely, the arm kinematics, hydrodynamic flow, structures, hand trajectory, and hand strike angles [11]. For kinematic parameters, the average swimming speed (v), stroke length (SL), and stroke rate (SR) act as indicators of the kinematic strategies used by each swimmer during a given task [12]. Additionally, the stroke index (SI) is recognized as an indirect measure of swimming efficiency [13,14]. SL can negatively affect swimming performance, while a small increase in the stroke rate can cause a significant increase in the total race time [15]. In long-duration events (>30 min), swimmers adopt a uniform profile throughout the race, selecting the swimming speed that they can maintain until the end of the competition, mainly, from the moment there is an accumulated muscular and psychological fatigue that causes the decrease of swimmer’s performance [7]. Recently, Puce et al. [9] showed a decrease in SL, which was offset by an increase in SR. These changes can also be explained through other factors associated with OWS, since it was found that wetsuit use during pool training might decrease swimming performance, namely, decrease the v and increase the core temperature [4].

Kinematic variables are closely associated with neuromuscular activity, as confirmed by increases in EMG amplitude and frequency, which reflect the greater muscular involvement during the swimming stroke [16].

Regarding the muscular analysis in OWS, a few studies have focused on understanding how swimmers maintain peak performance with the appearance of muscular fatigue, namely, in the swimming pool [9,10], through EMG analysis. Puce et al. [9] aimed to understand the effect of muscle fatigue in three different drafting positions (free, lateral, and behind swimming) and found that the behind swimming condition had lower muscle fatigue and higher swimming efficiency than free and lateral drafting. The muscle fatigue manifested a mean frequency (MNF) and an increase in the root mean square (RMS) value of EMG activity, showing a lower increase in RMS and lower decrease in MNF, which indicate a lower fatigue in the drafting configurations in comparison to free swimming. The muscles that showed the highest fatigue were the latissimus dorsi and triceps brachii, and the lower fatigue was observed in the lower limbs for the rectus femoris.

Further, for understanding the compensatory mechanisms used by swimmers during the performance of a controlled speed swimming test, they found that that there was a decrease in SL and SI, compensated by an increase in SF, and an increase in the muscular activity of almost all muscles, except for the muscle latissimus dorsi [10]. Moreover, Puce et al. [10], regarding the analysis of muscular activity for the stroke phases (propulsive and recovery), found that, for the upper limb muscles, there was a significant increase in activity in both phases of the stroke, except for the push phase in the biceps brachii and triceps brachii.

Based on the previously reported literature, it seems that scientific research is still needed to better understand kinematic and muscular activation in an OWS environment, because it is still scarce. Only a few studies have collected data in natural conditions such as lakes or the sea where the open water swimming races are developed, and the majority focus in pool conditions. Therefore, the main aim of this study was to analyse the kinematic parameters and neuromuscular activity in the OWS environment during a 5 km swim. Thus, it was hypothesized that (i) kinematic parameters such as SL, SI, and v will decrease, and SR will increase in the last part of the race; (ii) neuromuscular activity would differ between phases, with the higher muscular activation of recovery muscles (upper trapezius and anterior deltoid) and the progressive fatigue of propulsive muscles (latissimus dorsi, and triceps brachii); and (iii) environmental conditions inherent to open water environments may modulate these adaptations compared to pool settings.

## 2. Materials and Methods

### 2.1. Participants

Nine male open water swimmers (age: 25.4 ± 11.9 years; range: 15–42 years; body mass: 75.9 ± 9.0 kg; height: 180.7 ± 6.7 cm; and arm span: 185.6 ± 10.3 cm) with 371 ± 123 World Aquatic points at 1500 m event volunteered to participate in this study after being instructed about the procedures. Participants were only included if they met the requirements of having at least 7 years of experience and were participating in national competitions. In another case, they were excluded if they exhibited any health risk or condition that would affect OWS performance. Before the tests, the participants were informed of the benefits and risks of the investigation and signed an institutionally approved informed consent document. This study was approved by the University Ethics Committee and all procedures were performed in accordance with the Helsinki Declaration regarding human research.

### 2.2. Measures

Muscular activity was assessed on the left side of the body by surface electromyography (EMG) through a wireless EMG system with integrated accelerometers (Miniwave, Cometa, Milano, Italy; EMGandMotionsTools software 8.7.6.0) and memory-equipped probes of 7 g and a sample rate of 2000 Hz at 16 bits. For each subject, the skin under the electrodes was shaved, rubbed with sandpaper, and cleaned with alcohol so that the interelectrode impedance did not exceed 5 KOhm [17]. To isolate the electrodes from water, we used transparent dressings (Hydrofilm^®^, 10 cm × 1.5 cm, Rock Hill, SC, USA) and adhesive round gasket stickers to isolate the connection between EMG sensor and electrode [18].

The bipolar surface EMG Electrodes (Kendall ™, ECG electrodes, 57 × 34 mm) were placed parallel to the direction of the muscle fibres in the middle of the contracted muscle belly, along the longitudinal midline of the target muscle in accordance with the recommendations of the Surface Electromyography for the Non-invasive Assessment of Muscles project (SENIAM) guidelines for electrode placement [18], and the seven muscles analysed were upper trapezius (UT); latissimus dorsi (LD); pectoralis major (PM); posterior deltoid (PD); anterior deltoid (AD); triceps brachii (TB); and biceps brachii (BB), according to their importance in swimming propulsion.

Prior to the test, each subject performed a Maximal Voluntary Contraction (MVC) test on the left side, which is one of the most commonly used methods of EMG signal normalisation [19]. After the initial warming-up sequence, the MVC test consisted of each subject gradually increasing the force, reaching maximum effort within 3–5 s, holding it for 3 s, and calming down within 3 s. They repeat it at least once, with a rest of 30 to 60 s in between [19]. The MVC was compounded with a total of seven test positions, starting with UT. All the MVC tests were performed against a static resistance. When testing UT muscle, the swimmer should try to lift their arm upward against a static resistance that can be arranged by having a large-enough load pressing the shoulder down. Furthermore, it was important to ensure fixation of the elbow and trunk during the BB muscle testing. The same arrangement was used for maximal TB muscle contraction. In the case of LB muscle, adduction pressure was performed against resistance from 90 degrees of arm abduction [18].

Additionally, the swimmers wore their personal long-sleeved custom-made swimming suit, to protect the electrodes and sensors during the tests and to simulate competition conditions.

The EMG measurement was synchronised with a digital video camera (Panasonic, DC–FZ 1000II, Kadoma, Japan) positioned at 50 m from water level in an elevated position, to record the entire procedure. To synchronise the EMG data with the video, subjects had to stand on the water line in a T-Position for 5 s and then tap three times on the sensor located on AD before starting the trials. The same gesture of tapping three times on the AD muscle occurred when each subject finished the course.

### 2.3. Design and Procedures

**Figure 1:**
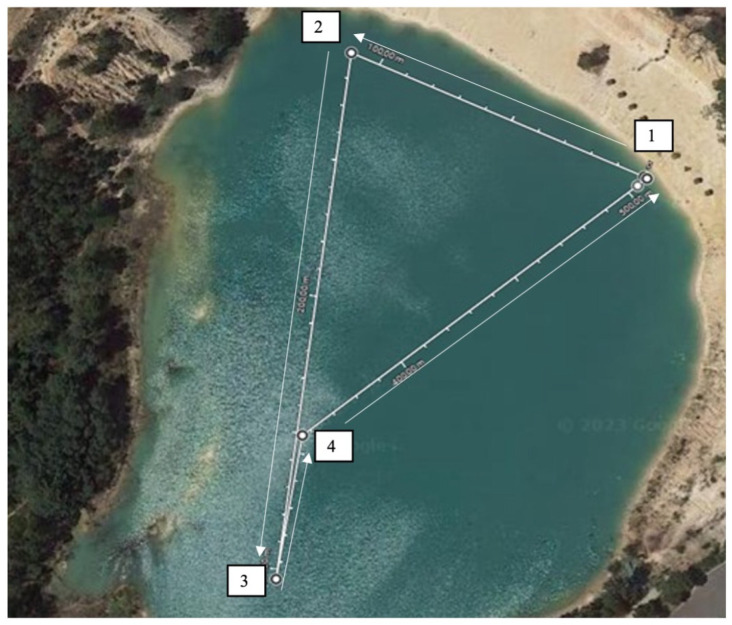
Course marked with four buoys and swimming direction. 1—start and end of the swimming; 2—swimmer turn left the buoy; 3—swimmer turn left the buoy; 4—swimmer turn right the buoy.

All the conditions were performed in the same location and before starting. The swimmers needed to swim in a pre-prepared course; the start and finish were on the same buoy. The protocol was made in lake where subjects had to swim on a course marked with four buoys (Figure 1). All procedures were performed in calm water, without current interference. The wind speed (15.7 ± 6.7 km/h), the air temperature (23 ± 4.7 °C), and water temperature (25 ± 0.8 °C) were recorded in each day of the test. These values fall within the acceptable range set by the Fédération Internationale de Natation (FINA), which defines that official open water swimming competitions must take place in water temperatures between 16 °C and 31 °C [19].

Before initiating the official trials, the subjects performed a 10-min warm-up like they usually do before every OWS race. Afterwards, each subject swam 5 × 1000 m, respecting their own competition strategies, with a 30 s check-in with the devices and to hydrate. There were four buoys and there was a line connecting the second and third buoy with a length of 45 m.

The final and split times for each swimmer were obtained by manual timing (3 × 100 m Stopwatch, Finis, Tracy, CA, USA) from the video recording and were taken for each trial. The mean speed (v) was calculated by the ratio of 45 m distance determined between the floats and the time spent. Stroke length (SL) [20] was calculated from the ratio between v and the corresponding SR Martens, and stroke index (SI) was calculated by multiplying v by SL [13].

Each video was synchronised with EMG signal in the software (EMG and Motion Tools). In this study, the synchronisation was processed by identifying peaks that were visible in the accelerometer signal, with an accuracy of 33.3 ms in one video frame.

Signal processing started with applying filters to the MVC file. EMG raw data were processed with the following filters: (i) low-pass filter with cut-off frequency of 400 Hz and a Butterworth filter of fourth order; and (ii) high-pass filter with cut-off frequency of 20 Hz and a Butterworth filter of fourth order. After signal rectification, Smoothing and Root Mean Square (RMS) envelope were used with a 50 ms window. The maximum MVC activation values (µV) for each muscle were then calculated and presented in relation to the percentage of MVC (% MVC). Finally, the last portion of processing was applying the same filters for the signal taken from each trial, as well as applying the MVC values to normalise the trial file. We made markings in the EMG files to signal the arm cycles and split the cycles into the underwater and recovery phase. The underwater phase started when the fingers began touching the water and the end of the cycle was when the fingers were almost out of the water. For the recovery phase, the start was when the fingers exited water, and the end was when the fingers started touching the water.

### 2.4. Statistical Analysis

SPSS statistical software version 28.0 (IBM Corp., Armonk, NY, USA) was used for statistical analyses. First, the normality plot tests of Shapiro–Wilk were applied, according to the sample size. After checking normality, the parametric ANOVA test for repeated measures was used for the kinematic parameters, and, to assess differences between distances, the paired-samples t-test was used. Together, the Partial Eta Squared (η^2^) was calculated as an indicator of the magnitude of the effect, with η^2^ < 0.3 considered a small effect; 0.3 < η^2^ < 0.5 a medium effect; and η^2^ > 0.5 a large effect [21,22]. In the electromyography data, we obtained a non-normal distribution, which required the use of a non-parametric test. The results were expressed in % MVC. Mann–Whitney U-tests were conducted on the non-normally distributed data to evaluate differences between cycle phases, and the Friedman test was used to detect significant differences among the observed parameters. Cohen’s d was calculated as an indicator of the magnitude of the effect, with D considered a small effect if <0.2; a medium effect if <0.5; and a large effect if >0.8. The statistical significance was set to *p* ≤ 0.05.

## 3. Results

Table 1 shows an increase in SR with a small effect (F = 1.54, *p* = 0.24, η^2^ = 0.16), especially in the final lap of the trial; a decrease in SL with a medium effect (F = 3.41, *p* = 0.06, η^2^ = 0.30), and a decrease in SI (F = 3.29, *p* = 0.08, η^2^ = 0.29) from the begin to the end of the trial.

It is noticeable that SL (F = 3.41, *p* = 0.06, and η^2^ = 0.30) and SI (F = 3.29, *p* = 0.08, and η^2^ = 0.29) changed along the distances covered, showing a decrease, essentially between 1000 m and 4000 m. Significant differences for SL between 1000 and 4000 m (*p* = 0.006), 2000 and 4000 m (*p* = 0.015), and 3000 and 4000 m (*p* = 0.005) were shown. Moreover, SI presented significant differences between 1000 and 2000 m (*p* = 0.006), 1000 and 3000 m (*p* = 0.003), and 1000 and 4000 m (*p* = 0.001), contrary to SR (F = 1.54, *p* = 0.24, and η^2^ = 0.16), which increased, an increase especially in the last 1000 m (32.5 ± 3.0 cycles-min^−1^) when compared to the other laps. Particularly, SR had decreased from 1000 to 3000 m (*p* = 0.05) and showed an increase from 3000 to 4000 m, with significant differences.

### Eletromyography

Regarding the muscle patterns of the swimming cycle, we observed higher activation in TB during the underwater phase, and UT during the recovery phase. Table 2 presents the significant differences in muscular activity between the phases along the distance.

The muscles AD, TB, and LD showed significant differences between phases throughout the distance. UT showed significant differences (*p* = 0.00) with a large effect between the underwater (13.25% MVC) and recovery phases (31.25% MVC) from 4000 m to 5000 m.

The AD muscle is highly activated in recovery, showing statistical differences from the beginning (*p* = 0.01) to the end of the race (*p* = 0.03), with a large effect. It is also noted that there is a difference between the underwater (6.55% MVC) and recovery (16.46% MVC) phases, with higher activation at 1000 m, contrary to the TB muscle, which is mostly recruited in the underwater phase (22.80% MVC), from the start (*p* = 0.00) to the finish (*p* = 0.03), showing a significant difference in each lap, with a large effect. In terms of the LD, there were significant differences in muscle activation, from 1000 m (*p* = 0.00) with a huge effect, to 5000 m (*p* = 0.01) with a large effect. The LD muscle showed a higher recruitment in the underwater phase (10.51% MVC) compared to the recovery phase (2.96% MVC) at the start of the trial, slowly decreasing throughout the distance.

## 4. Discussion

The aim of the study was to characterise and analyse the kinematic parameters and neuromuscular activity of OWS during a 5 km swim.

The key findings of this study indicate that, for the kinematics, SR increased through the 5000 m race, with a higher increase at 4000 m, the swimming speed decreased at 2000 m and then stabilised at 3000 m and increased in the last 1000 m, the SI was always decreasing until 4000 m with a slight increase in the last 1000 m, and there was a tendency for the SL to decrease to maintain performance in the last 1000 m. Regarding the neuromuscular activity, the higher muscle activation were in the UT and AD muscles (recovery phase) and TB and LD muscles (underwater phase). The UT in the recovery phase and the TB in the underwater phase were the muscles with a higher muscle activation during all of the 5 kms. Regarding the stroke phases of the swimming cycle, for the recovery phase, the higher muscle activation was observed in UT, PD, AD, and BB and, for the underwater phase, in TB, PM, and LD.

In this way, the hypotheses of this study—that kinematic parameters such as SL, SI, and v will decrease, and SR will increase in the last part of the race; and neuromuscular activity will be higher in the recovery phase—were confirmed.

### 4.1. Kinematic

The kinematical variables v, SR, SL, and SI have been used to analyse the swimmer’s technical skill level, with the evaluation of these factors considered to be important for training planning and describing swimmers’ performance [12].

There is a gap in the literature, since few studies have analysed kinematical variables in natural conditions in OWS and with open water swimmers. The study of Zacca et al. [2] simulated, in pool conditions and in open water conditions, the 5 × 1000 m front crawl for open water swimmers; otherwise, the study presented by Lopez-Belmonte et al. [5] compared the 1500 m swim in pool and open water conditions in triathletes. Our findings are closer to the results presented by Zacca et al. [2], which can be due to the fact that both studies used a very similar protocol and because the competitive level of the swimmers are very similar, while, compared to the results shown by Lopez-Belmonte et al. [5], our results are slightly lower, perhaps due to differences in the protocol and competitive level of the triathletes.

In a competitive context, swimmers with a better swimming technique seem to have a greater capacity to maintain SL [20,23,24]. According to the results obtained in our study, it is possible to verify that, of the biomechanical variables analysed, we observed a progressive increase in SR and a decrease in SI, results that are in line with previous studies [6,25], which can be explained through the biomechanical readjustments made by the swimmer to long-lasting tasks with moderate intensities due to the onset of fatigue.

The attempt to increase the stroke length (SL) around 4000 m to improve velocity may have paradoxically led to a reduction in SL, triggering an increased muscular effort in some muscle groups and compensatory activation in others in response to fatigue.

According to Pelarigo et al. [6], the accumulation of fatigue is related to the intensity and duration of the exercise by causing biomechanical changes in swimmers, leading to readjustments during the race. Furthermore, the stabilisation of v stands out, which can be justified by the swimmer adopting a uniform profile throughout the race, selecting the v that he can maintain until the end [7]. However, the competitive nature of the swimmers and the objective lead to an increase in SL so that an increase in v occurs, essentially, in the final stages (4000 m). According to Baldassarre et al. [1], it was identified that OW swimmers with better results use the strategy of increasing v in the final part of the competition, which is in line with the results obtained. Not only does increasing SL aim to increase v, but it also reduces SL, as the swimmer tends to perform more stroke cycles to maintain performance [7].

### 4.2. Neuromuscular Activity

Regarding the analysis of neuromuscular activity, all the results of the studies presented above were measured in pool conditions, because this study represents a novelty in this field.

Therefore, there was higher muscle activation for the UT and AD muscles in the recovery phase of the swimming stroke and in the underwater phase for the TB and LD muscles. When comparing our results with [26], we can observe that LD and TB differ between the underwater and recovery phase, since it was shown that these muscles are mostly recruited in underwater phase. On the other hand, ref. [27] concluded that, in front crawl, the muscles with higher muscle activation during the swimming phases were the PM and LD, which can be considered as the main propulsive muscles of the front crawl. PM had a muscular activation of 71% of MVC in the initial phase of the underwater phase and LD (peaked at 75% of MVC) in the final underwater phase when swimming at a moderate pace. In the study [28], the LD was different because, in this study, an average of 3.2% MVC was obtained, which was very different from 44.81% MVC.

When analysing other muscles, we observed non-significant differences for the PM, as it presents similar activation patterns in both phases. Once again, for the LD, significant differences were found between the stroke phases, which shows that the LD is highly active, especially in the underwater phase. In the study by [29], the AD muscle shows the lowest activity in the final underwater phase of the stroke cycle, showing the same behaviour throughout the test in our study, mainly having a tendency to decrease between each 1000 m. Some changes in muscle participation were detected during the swimming courses, which were more evident from 4000 m onwards. Lauer et al. [30] continued the study by [29], and examined the muscle activation patterns of the BB and TB muscles in depth EMG. According to front crawl studies focused on the EMG amplitude in the muscles [26,28], LD and TB are most involved in the underwater phase. In fact, the LD is known due to its important role in the successful completion of each of the swimming strokes [31] and, together with the TB, is considered the key muscle in maintaining speed.

When comparing our results to recent studies, notable similarities emerge, particularly regarding the reduction in LD activity during the underwater phase of the stroke cycle [9,10,32]. The results from [9] and our study showed a strong relationship in terms of muscle recruitment during swimming. For example, [9] crowned LD the “swimmer muscle”, due to its crucial role in the successful execution of all swimming styles. Together with TB, LD recruitment was considered crucial for maintaining speed [33]. This clearly aligns with our results, since we observed that TB and LD were more activated during the underwater phase, which can be explained according to the movement stabilisation required to overcome water resistance in the underwater technique. In addition, in our study, we found that UT and AD were the muscles that had higher activation in the recovery phase, with UT being particularly recruited during this phase. Once again, this result is very similar to the findings of [9,10]’s research, since these muscles contributed significantly to upper limb movement control during the recovery phase. Moreover, the results are in line with the study developed by [32]: since the AD muscle is activated in the initial recovery phase of the stroke, swimmers can reduce their time in the recovery phase to reach a faster pace with a simultaneous increase in muscle activity. Furthermore, it was reported that participants activated their PD muscle more during the underwater phase period in order to swim faster. Our results are very similar to the study by [30] as they obtained results where BB is more recruited during recovery and TB during the final phase of the front crawl.

Environmental conditions are known to influence both physiological strain and biomechanical performance in open water swimming. In this study, the water temperature of approximately 25 °C, along with a moderate air temperature and wind speed, provided favourable conditions for long-distance swimming. According to FINA regulations, races must occur within a 16–31 °C water temperature range to minimise thermal stress and ensure athlete safety [19]. Although the observed parameters were within these acceptable limits, recent evidence suggests that water temperatures above 23–25 °C may still impose significant thermoregulatory challenges. Heat-related illnesses—including exertional heat exhaustion and near-syncope—have been documented in open water swimmers under such conditions, particularly when combined with conditions of high humidity, low wind, or inadequate hydration [34]. These factors may silently increase physiological strain even in seemingly “safe” environments, especially during prolonged efforts.

Even under moderate thermal conditions, sustained physical exertion can contribute to cumulative neuromuscular fatigue, fluid loss, and compensatory changes in stroke technique. Such fatigue-induced adaptations particularly in stroke coordination and muscle recruitment can elevate the risk of overuse injuries, such as impingement syndrome or rotator cuff tendinopathies. Therefore, the environmental context must be considered alongside fatigue progression when interpreting the performance data and assessing the injury risk in both competitive and recreational open water swimmers.

In sum, our study suggests that significant changes in the kinematic parameters of the stroke occurred particularly after 4000 m, where swimmers showed a decrease in SL and a slight increase in SI, likely due to the increase in v and SR. This indicates that the distance covered per stroke cycle shortened every 1000 m, potentially linked to the onset of fatigue. As swimmers fatigue, changes in kinematic parameters such as SL and SR may be a result of altered muscle activation patterns aimed at compensating for reduced energy levels. The observed muscle activation patterns, as reflected in the EMG data, seem to align with these kinematic changes. The UT and AD muscles showed higher activity during the recovery phase, which corresponds to the need for more upper limb control, stabilisation, and overcoming water resistance. We assume that increased activations of specific muscles likely help swimmers maintain stroke efficiency despite the decreasing distance covered per cycle. Thus, the changes in kinematic variables such as SL, SR, and SI appear to be closely linked with specific shifts in muscle activation to adapt to fatigue during prolonged swimming efforts. It seems that, by learning to manipulate their SL and SF, and eventually their arm coordination, swimmers can achieve a given velocity with a lower energy cost of swimming and higher muscular activation.

### 4.3. Practical Applications

The findings highlight the need for training programs that prioritise muscle groups most involved during the underwater and recovery phases of the stroke, particularly in the upper limbs, which are particularly susceptible to overuse injuries due to repetitive loading, and high training volumes, which increase the stress on muscle, tendons, and joints—in this context, implementing a periodised plan, for example, dry-land training focused on strength training, especially for the shoulders and core, and developing the neuromuscular coordination to enhance muscular efficiency and resilience. Such training should also include flexibility, mobility, and scapular stabilisation exercises, which are known to reduce the incidence of shoulder-related injuries in swimmers.

Monitoring fatigue through surface electromyography further supports this approach by identifying the most fatigued muscle groups, enabling targeted prevention strategies. Importantly, the longitudinal monitoring of fatigue progression—such as changes in the EMG amplitude (RMS) and frequency content over time—can help detect early signs of neuromuscular decline, allowing for timely training adjustments and recovery interventions.

Notably, fatigue-related changes in stroke biomechanics and kinematics may elevate the risk of impingement syndrome and other musculoskeletal injuries, particularly in both competitive and recreational open water swimmers [27,35,36]. These findings are not only relevant for elite athletes but can also inform coaching practices and public health recommendations for safe participation in long-distance swimming. Implementing individualised recovery protocols and workload management based on fatigue trends may further reduce injury risk and optimise long-term performance.

### 4.4. Limitations

Some limitations of this study should be addressed. First, expanding the sample size to include a wider range of experience levels and genders, along with exploring performance differences, would provide a more comprehensive understanding of kinematic and neuromuscular activity during open water swimming events. Additionally, a larger sample with greater variability would improve the robustness of the findings. Second, the protocol for the 5 × 1000 m swim included four brief 30-s pauses, which may not fully replicate a continuous 5 km race. Third, data collection occurred on different days due to weather conditions, which could have introduced variability. Moreover, muscular activity in the lower limbs was not measured because of the device limitations affecting EMG data collection. Moreover, the absence of other swimmers during each test does not fully replicate real open water swimming events.

Future studies should analyse the activity of the upper and lower limb muscles on both sides of the body to better understand the contribution of the upper and lower limbs to the muscular effort, and to assess the impact of head orientation when turning around the buoys during the course. Incorporating additional physiological markers, such as the rating of perceived exertion, heart rate monitoring, and blood lactate concentrations, could further improve the control of intensity and the detection of fatigue.

## 5. Conclusions

The analysis of EMG and the kinematic parameters provided valuable insights into the mechanisms swimmers use to cope with the demands of long-distance open water races. From a kinematic perspective, most changes occurred around 4000 m, including an increase in the stroke rate (SR) as swimmers attempted to maintain velocity in the final, decisive stage. Neuromuscular activity also showed significant phase-dependent variations throughout the trial. The differences in muscle recruitment between the recovery and underwater phases reflected the effort required to overcome drag and stabilise the body position.

These findings reveal the fatigue-related adaptations in both movement and muscle activation, which may contribute to overuse injuries particularly in the shoulder complex. While the results are relevant for trained swimmers, they also provide valuable guidance for recreational athletes, who may be more vulnerable due to limited technical support. As open water swimming continues to grow in popularity, these insights support the development of safer, evidence-based training approaches that address fatigue and injury risk across all levels of participation.

## Figures and Tables

**Table 1:** Mean ± standard deviation (Mean ± SD) for kinematic variables (v, SR, SL, and SI) through the distance covered by the swimmers. *p*-values and effect sizes are also shown.

	1000 m	2000 m	3000 m	4000 m	5000 m		
Kinematic Variables	Mean ± SD	Mean ± SD	Mean ± SD	Mean ± SD	Mean ± SD	F-Ratio (*p*)	η^2^
v [m/s]	1.39 ± 0.18	1.38 ± 0.15	1.38 ± 0.14	1.37 ± 0.16	1.39 ± 0.18	1.94 (0.18)	0.19
SR [cycles-min^−1^]	31.4 ± 3.04	31.1 ± 3.88	31.2 ± 3.29 ^f^	32.1 ± 2.76	33.0 ± 3.36	1.54 (0.24)	0.16
SL [m-cycle^−1^]	2.72 ± 0.35 ^c^	2.67 ± 0.34 ^d^	2.65 ± 0.33 ^e^	2.60 ± 0.33	2.58 ± 0.40	3.34 (0.06) *	0.29
SI [m^2^s^−1^cycle^−1^]	3.90 ± 0.82 ^abc^	3.71 ± 0.78	3.68 ± 0.75	3.61 ± 0.83	3.64 ± 0.94	3.29 (0.08) *	0.29

Key: *—The statistical significance was set (*p* ≤ 0.05) between 1000 and 2000 ^a^, 1000 and 3000 ^b^, 1000 and 4000 ^c^, 2000 and 4000 ^d^, 3000 and 4000 ^e^, and 3000 and 5000 ^f^; v—velocity; SR—Stroke Rate; SL—Stroke Length; and SI—Stroke Index; m/s—meters per second; cycles-min^−1^—cycles per minute; m-cycle^−1^—meters per cycle; m^2^s^−1^cycle^−1^—product of stroke length with velocity.

**Table 2:** Mean ± SD of the % MVC for the comparison between the underwater and recovery phases along the distance, on the left side of the body during all seven studied muscles. *p*-values and effect sizes are also shown.

Distance		1000 m			2000 m			3000 m			4000 m			5000 m		
Muscles	Phase	Mean ± SD	*p*-Value	d	Mean ± SD	*p*-Value	d	Mean ± SD	*p*-Value	d	Mean ± SD	*p*-Value	d	Mean ± SD	*p*-Value	d
UT	UW	14.96 ± 15.09	0.06	−0.78	14.74 ± 6.78	0.02 *	−1.15	15.44 ± 9.89	0.02*	−1.23	13.92 ± 9.08	0.01*	−1.22	13.25 ± 7.32	0.00 *	−1.41
	REC	37.46 ± 38.09			32.18 ± 20.29			34.28 ± 19.28			30.73 ± 17.19			31.25 ± 16.57		
PD	UW	16.98 ± 4.63	0.51	−0.41	12.58 ± 8.59	0.89	0.31	8.93 ± 4.99	0.69	0.03	11.86 ± 6.77	0.97	−0.11	11.60 ± 7.80	0.97	−0.16
	REC	19.56 ± 7.52			10.41 ± 4.63			8.77 ± 6.77			12.77 ± 9.29			13.13 ± 10.75		
AD	UW	6.55 ± 5.85	0.01 *	−1.16	4.72 ± 3.79	0.04 *	−1.08	4.88 ± 3.99	0.04 *	1.07	3.49 ± 2.20	0.02 *	−0.98	3.75 ± 2.58	0.03 *	−0.93
	REC	16.46 ± 10.55			11.84 ± 8.54			12.22 ± 8.89			9.47 ± 8.36			9.37 ± 8.14		
BB	UW	11.55 ± 3.40	0.31	−0.55	11.01 ± 4.15	0.31	−0.49	10.43 ± 3.24	0.17	−0.79	10.04 ± 2.78	0.20	−0.69	10.03 ± 2.61	0.27	−0.67
	REC	15.31 ± 9.09			13.84 ± 7.13			15.52 ± 9.64			14.25 ± 8.11			13.19 ± 6.19		
TB	UW	22.80 ± 7.10	0.00 *	2.02	21.19 ± 7.53	0.00 *	1.84	19.71 ± 6.87	0.00 *	1.74	18.80 ± 8.09	0.01 *	1.56	19.16 ± 9.09	0.03 *	1.24
	REC	10.22 ± 5.24			8.97 ± 5.60			9.04 ± 5.26			8.52 ± 4.60			9.87 ± 5.44		
PM	UW	15.20 ± 8.33	0.12	0.74	13.13 ± 6.14	0.06	0.93	13.30 ± 6.12	0.09	0.86	12.12 ± 5.18	0.15	0.77	12.02 ± 5.26	0.45	0.30
	REC	9.21 ± 7.97			8.28 ± 4.13			8.63 ± 4.71			8.33 ± 4.63			10.12 ± 7.12		
LD	UW	10.51 ± 4.36	0.00 *	2.34	9.87 ± 4.67	0.00 *	2.01	9.75 ± 4.48	0.00 *	2.10	9.00 ± 4.04	0.01 *	1.74	8.91 ± 3.91	0.01 *	1.57
	REC	2.96 ± 1.35			2.94 ± 1.37			2.77 ± 1.50			3.30 ± 2.26			3.83 ± 2.38		

Key: *—The statistical significance was set to *p* ≤ 0.05; UW—Underwater; REC—Recovery; Mean ± SD—Mean ± Standard Deviation; and d—Cohen’s d Effect Size.

## Data Availability

The original contributions presented in the study are included in the article, further inquiries can be directed to the corresponding author.
